# A practical approach to detect ancestral haplotypes in livestock populations

**DOI:** 10.1186/s12863-016-0405-2

**Published:** 2016-06-24

**Authors:** Enrique Sánchez-Molano, Dimitrios Tsiokos, Dimitrios Chatziplis, Hossein Jorjani, Lorenzo Degano, Clara Diaz, Attilio Rossoni, Hermann Schwarzenbacher, Franz Seefried, Luis Varona, Daniele Vicario, Ezequiel L. Nicolazzi, Georgios Banos

**Affiliations:** The Roslin Institute and Royal (Dick) School of Veterinary Studies, University of Edinburgh, Easter Bush, Midlothian, EH25 9RG Scotland UK; Laboratory of Agrobiotechnology and Inspection of Agricultural Products, Department of Agricultural Technology, School of Agricultural Technology, Food Technology and Nutrition, Alexander Technological Educational Institute of Thessaloniki, Thessaloniki, Greece; Interbull Center, Uppsala, S-75007 Sweden; Associazione Nazionale Allevatori Bovini di razza Pezzata Rossa Italiana, Udine, Italy; Departamento de Mejora Genética Animal, INIA, Madrid, 28040 Spain; Associazione Nazionale Allevatori Bovini della Razza Bruna, Verona, Italy; ZuchtData EDV-Dienstleistungen GmbH, Wien, Austria; Qualitas AG, Zug, Switzerland; Departamento de Anatomía, Embriología y Genética, Universidad de Zaragoza, Zaragoza, 50013 Spain; Instituto Agroalimentario de Aragón (IA2), Zaragoza, 50013 Spain; Bioinformatics core facility, Fondazione Parco Tecnologico Padano, Via Einstein, Loc. CascinaCodazza, Lodi, 26900 Italy; SRUC,The Roslin Institute Building, Easter Bush, Midlothian, EH25 9RG Edinburgh UK; School of Veterinary Medicine, Aristotle University of Thessaloniki, Thessaloniki, Greece

**Keywords:** Ancestral haplotypes, Population dynamics, Selection signatures, Cattle

## Abstract

**Background:**

The effects of different evolutionary forces are expected to lead to the conservation, over many generations, of particular genomic regions (haplotypes) due to the development of linkage disequilibrium (LD). The detection and identification of early (ancestral) haplotypes can be used to clarify the evolutionary dynamics of different populations as well as identify selection signatures and genomic regions of interest to be used both in conservation and breeding programs. The aims of this study were to develop a simple procedure to identify ancestral haplotypes segregating across several generations both within and between populations with genetic links based on whole-genome scanning. This procedure was tested with simulated and then applied to real data from different genotyped populations of Spanish, Fleckvieh, Simmental and Brown-Swiss cattle.

**Results:**

The identification of ancestral haplotypes has shown coincident patterns of selection across different breeds, allowing the detection of common regions of interest on different bovine chromosomes and mirroring the evolutionary dynamics of the studied populations. These regions, mainly located on chromosomes BTA5, BTA6, BTA7 and BTA21 are related with certain animal traits such as coat colour and milk protein and fat content.

**Conclusion:**

In agreement with previous studies, the detection of ancestral haplotypes provides useful information for the development and comparison of breeding and conservation programs both through the identification of selection signatures and other regions of interest, and as indicator of the general genetic status of the populations.

**Electronic supplementary material:**

The online version of this article (doi:10.1186/s12863-016-0405-2) contains supplementary material, which is available to authorized users.

## Background

The availability of whole-genome genotyping platforms such as the Illumina BovineHD chip containing 777,962 Sinlge Nucleotide Polymorphsism (SNP) [1] has facilitated genomic selection and prediction methods for genetic improvement of livestock [2], but also provided useful tools to study the evolution and dynamics of genetic variation [3] and to untangle the history of populations [4]. These high density SNP arrays provide a dense coverage of the entire genome, thus allowing the precise detection and mapping of regions associated with traits of interest or regions with specific characteristics such as recombination hotspots or runs of homozigosity.

In response to population size reduction, selection, drift and/or other evolutionary processes, particular combinations of alleles can be conserved over many generations more often than expected by chance, leading to the development of linkage disequilibrium (LD) blocks. The length of these co-inherited genetic blocks, known as haplotypes, is proportional to the level of LD across the genome, and their study becomes crucial in order to understand the dynamics and characteristics of populations including selection signatures, recombination hotspots and bottlenecks.

However, before haplotypes can be detected, raw genotypes have to be analysed in order to determine which one of the pair of chromosomes holds each allele (phase). Different methods have been developed in this respect using genotypes based on single nucleotide polymorphisms (SNP): Expectation-maximization [5, 6], coalescent models [7], Monte Carlo approaches [8], identity by descent (IBD) probabilities [9], and long range phasing and library imputation methods [10, 11]. Although in many of these methods the phasing accuracy depends mainly on the sample size, marker density and population structure [12], in other cases only the sample size affects their performance [10].

The interest of ancestral segregating haplotypes is multiple: Regional association mapping studies can be performed in order to detect causative variants related to quantitative animal traits of interest [13], thus providing greater power than simple SNP-based genome-wide association analyses when LD is extensive [14–17]. Furthermore, the study of the frequency distribution of ancestral segregating haplotypes provides information about population dynamics such as bottlenecks and adaptation [18]. Ancestral haplotype frequencies are expected to vary due to perturbations in the population, leading to a potential deficit of some haplotypes after moderate bottlenecks [19] and/or to unusual high frequency of specific haplotypes in certain sub-populations due to genetic drift and/or selection. Selective sweeps resulting from recent intensive selection lead to extended LD patterns and long highly frequent haplotypes [20–23], whereas old selection is expected to lead to shorter haplotypes as a consequence of recombination breaking the original blocks over the generations. Moreover, additional genomic characteristics such as recombination hotspots can also be detected [24, 25], as these genomic regions show a higher haplotype variation than expected under neutral theory.

In cattle, intense selective breeding over the past decades has led to a severe reduction of the effective population size [26] and, therefore, to an increase in inbreeding. This has led to certain phenotypic uniformity within breeds, but has also proven to be related to negative effects such as an increase in fertility and health problems due to inbreeding depression [27]. This intensive selection has also increased the frequency of favourable alleles for the traits of interest, leading to the establishment of strong but different LD patterns across breeds [28, 29]. Such breeds may have diverged based on their appearance, performance and/or geographical origin, and have been maintained through within-breed selection or with different degrees of admixture in order to further increase their performance. Due to their extended LD, the analysis of the ancestral haplotype diversity is expected to clarify the evolutionary history of these breeds. Ancestral haplotypes may also allow the identification of possible introgressed genomic regions related to conservation and of common regions under selection potentially affecting production or fitness related traits.

Therefore, the main objective of this study was to develop a procedure to detect ancestral haplotypes based on whole-genome SNP scanning that are segregating across several generations, both within and between breeds with genetic links. Previous studies have searched for specific ancestral haplotypes as candidate regions associated with particular traits. A genome-wide approach was taken in the present study with no trait or regional restriction applied. The developed algorithm was tested on simulated data and then applied to real data from Fleckvieh, Simmental, Brown Swiss and Spanish cattle breed populations. Examination of real data led to the detection of common selected regions among breeds and demonstrated haplotype diversity patterns concordant with the evolutionary history of the different populations.

## Methods

### Simulated data

Simulated data was created using a modified version of the simulation software GenoSim [30], to include the possibility to simulate multiple populations with variable selection intensity, but all coming from a common (base) population [31]. A base population of 400 animals (200 males and 200 females) was simulated for 40,000 generations under random mating and equal contributions to achieve the mutation-drift balance, as in the Fisher-Wright population model [32], with expected allelic frequencies of 0.5. In generation 40,000, two breeds, each with 200 animals (100 males and 100 females), were created from this base population sharing 50 % of ancestors in the base generation (*G0*). In later generations (*G1*-*G10*), each breed was independently maintained under phenotypic selection, with 30 % of males and 80 % of females being selected as parents of the next generation [31].

Simulated genomes consisted of 30 chromosomes of equal length (1 Morgan) with 52,830 evenly distributed SNPs. All polymorphisms had been generated during the 40,000 generations of the Fisher-Wright population model, assuming a mutation rate of 10^-4^ per nucleotide and a number of recombination events per chromosome and generation sampled from a Poisson distribution with mean of 1. Using the formula described by Goddard [33] and based on the number of independent chromosome segments and the effective size, a proportion (30 %) of the non-monomorphic SNPs (Minimum Allele Frequency >0.05) was randomly assigned to be a functional gene (QTL).

Three different traits with heritabilities *h*_1_^2^ = 0.1; *h*_2_^2^ = 0.25; *h*_3_^2^ = 0.8 and phenotypic variances equal to 1 were simulated, being genetically correlated (*r*_12_ = 0.5; *r*_13_ = − 0.5). The assumed parameters render the simulated traits representative of a wide range of economically important animal traits. Simulated additive QTL effects for these traits were drawn from a normal distribution *N* ~ (0, *α*^2^) with *α* being the average effect of allelic substitution $\left(\sqrt{Va/2npq}\right.$ where *n is* the number of loci affecting the trait, *Va* is the genetic variance of the trait and *p* and *q* are the allelic frequencies with starting value of 0.5 [32]).

### Real data

Three different datasets were studied including, i) seven Spanish beef breeds, ii) two Fleckvieh/Simmental dual purpose cattle populations and iii) five Brown Swiss dual purpose cattle populations. In all cases, animals were genotyped with the Illumina BovineHD chip containing 777,962 SNPs [1]. Quality control (Additional file 1) was applied independently to each dataset using PLINK 1.07 [34] in order to assure sample and marker quality. Genomic quality control was applied by considering only autosomic loci with a call rate higher than 0.95. In addition, sample quality control was applied by removing animals with a call rate lower than 0.95. As suggested in other relevant studies [3], alleles with low frequency can provide useful information on diversity, and therefore, no Minor Allele Frequency threshold was applied in the present study.

The seven Spanish breeds included Asturiana (as), Avileña (av), Bruna (br), Morucha (mo), Pirenaica (pr), Retinta (re) and Rubia Gallega (rg). Each breed provided 25 trios (sire-dam-offspring. 75 animals per breed) genotyped 735,239 SNPs after quality control and some trios removed due to bad DNA quality (Additional file 1). In this case, SNPs with Mendelian errors greater than 0.095 were removed from further analyses. These breeds are autochthonous populations of beef cattle. After domestication, three main bovine groups (Turdetanus trunk, Iberian trunk and Cantabrian trunk) were established in Spain according to geographical preferences and phenotypic characteristics mostly related to coat colour. The Turdetanus trunk, including the Bruna, Pirenaica and Rubia Gallega breeds of the present study, originated in Asia Minor, was introduced to Africa and Europe via Egypt and is currently distributed across Andalusia, Galicia and Pyrenees. The Iberian trunk, including the Morucha and Avileña breeds, was introduced from the north of Europe through Celtic migrations and is mainly distributed in the centre of the Iberian Peninsula. The Cantabrian trunk, including Asturiana, probably descended from the ancient local bovine populations existing before the arrival of the indoeuropean cattle and is mainly distributed in the north of Spain. Retinta has been suggested to be an intermediate breed between the Iberian and the Turdetanus trunks.

The Fleckvieh/Simmental dataset consisted of 490 genotyped bulls from Austria, Italy, Germany and Switzerland, of which 473 bulls and 714,759 SNPs remained after quality control. In this dataset, Fleckvieh and Simmental were considered to be two independent populations with their own pedigree structures and including 315 and 158 genotyped bulls, respectively. Simmental cattle is a dual-purpose breed that was originated in western Switzerland as a result from the crossing between German and indigenous Swiss stocktracing back to the Middle Ages. This breed has been gradually exported globally, reaching Italy in the XV century, Eastern Europe and Africa in the XIX century, and the American continent in the XX century. Crossings of Simmental with autochthonous cattle has led to other synthetic breeds. This is the case of Fleckvieh, resulting from the cross of Swiss Simmental cattle with local Bavarian breeds in the XIX century. Fleckvieh, is considered a dual-purpose breeds and may be found in several countries such as Germany, Spain, Belgium, Hungary, Paraguay and Peru.

The Brown-Swiss dataset consisted of a total of 417 genotyped bulls from five different countries, of which 412 bulls and 714,759 SNPs remained after quality control. In this dataset, each country was considered as an independent population with its own pedigree structure: Austria (21 bulls), Germany (54 bulls), Italy (77 bulls), Switzerland (184 bulls) and USA (77 bulls). The Brown-Swiss breed is one of the oldest breeds, originating in Swiss Alps about 4000 B.C. Records from the Einsiedeln Monastery (Schwyz canton in Switzerland) indicate that Brown-Swiss breeding was already performed in the XIV century, being extended to Germany and other areas of the Alps. In the late XIX century, a few animals from this breed were exported to USA from the Schwyz canton, with subsequent exportations being performed and with the first cow and bull being recorded in the American herd book in 1880. In 1906, Brown-Swiss was declared a breed in USA and started to be heavily selected for milk production and other dairy characteristics, while in Switzerland little selection was done at the time, with no herd books being maintained until 1911. In the 1960s, the improved genetics of the American Brown-Swiss were exported back into Europe and crossed with the existing herds of European Brown-Swiss, leading to the establishment of upgraded Brown-Swiss populations in Germany, Italy and Austria.

### Genotype phasing and cluster definition

After quality control, genotypes were phased within each dataset (simulated and real). Genotype phasing and cluster definition were performed using AlphaPhase 1.1 [35], a software based on the Extended Long Range Phasing and haplotype Library Imputation methods [11, 36]. Genotypes were partitioned in clusters, using cores (haplotypes) of 100 SNPs with additional tails of 100 SNPs to each side of the core in order to properly define surrogates (i.e. relatives that share a region IBD with the animal and can be considered parents when carrying parental haplotypes of the animal [11]). These cores were not allowed to overlap and general parameters were set up as recommended [11], with the requirement that 10 surrogates should be considered before declaring a phase. A maximum of 10 % of surrogate conflict and 1 % SNP errors (including both inconsistencies and missing SNP) were allowed per core.

### Analysis within population

Based on the phased genotypes, haplotypes of the base generation (*G0* in the simulated data or the oldest generation with genotypes in the real data) were identified in every cluster and considered to be ancestral, estimating their frequency. In subsequent generations, the frequency of ancestral haplotypes and their similarity matrix (based on their SNP genotypes) was computed in every cluster. Ancestral haplotype frequencies and similarities were then averaged across all clusters, thus providing the average frequency of ancestral haplotypes and the average similarity between ancestral haplotypes across the entire genome. The latter is also an estimate of the molecular co-ancestry.

### Analysis among populations

In order to investigate the evolutionary history of Fleckvieh/Simmental and Brown-Swiss (Spanish breeds were not considered given the lack of pedigree depth), the ancestral haplotypes identified in every cluster within a given population were also traced in the other populations of the same dataset to estimate the percentage of haplotypes shared between populations.

Observed trends for co-ancestry and proportion of segregating ancestral haplotypes through generations were analysed using simple linear regression on number of generations.

## Results

### Simulated data

**Fig. 1:**
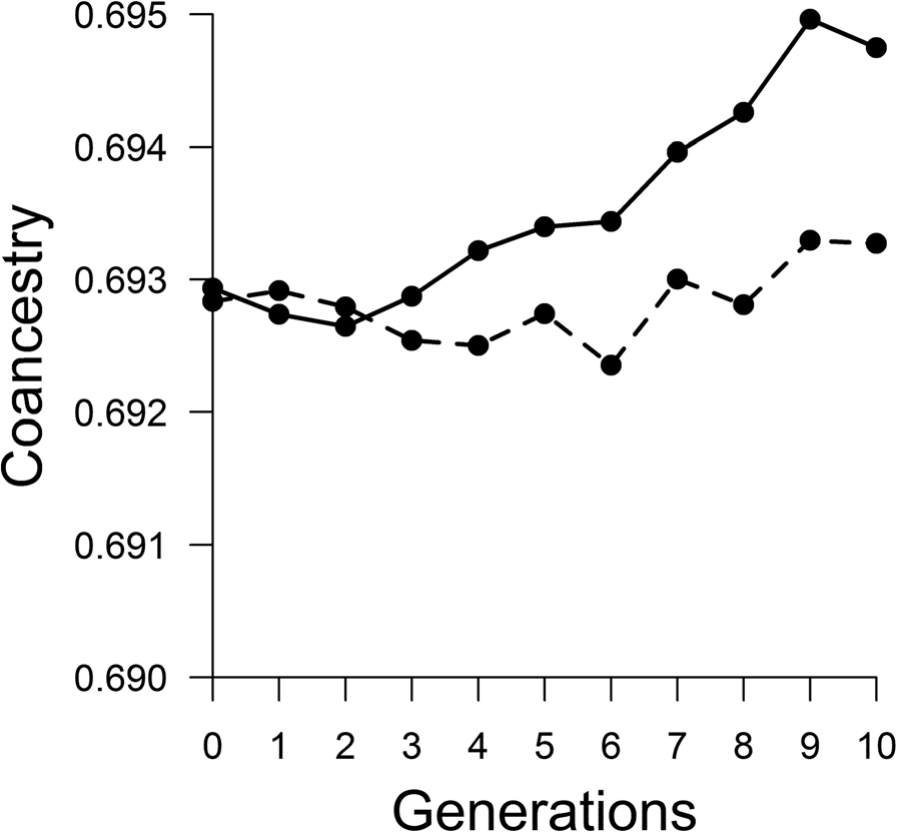
Similarity (co-ancestry) between ancestral haplotypes across generations in simulated dataset: Solid line corresponds to population 1 and dashed line to population 2

**Fig. 2:**
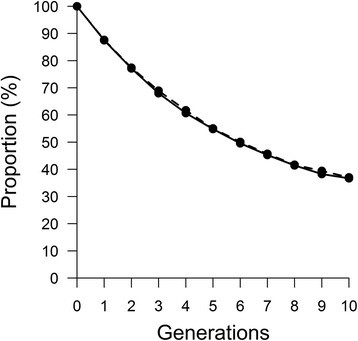
Proportion of ancestral haplotypes segregating per generation in simulated dataset: Solid line corresponds to population 1 and dashed line to population 2

A total of 528 clusters were obtained during phasing and analysed. The two simulated breeds presented similar haplotype variability (i.e. number of haplotypes per cluster across all generations) with averages of 344.35 and 342.42 haplotypes observed per cluster respectively. The change of the molecular co-ancestry for ancestral haplotypes through generations (Fig. 1) showed an increasing trend concordant with the expected effects of selection and drift. However, the trend magnitude (i.e. regression slope) differed in the two populations (*b*_*A*_ = 2.20×10^-4^ and *b*_*B*_ = 4.12×10^-5^), probably due to the random sampling of haplotypes caused by genetic drift. Similarly, the proportion of ancestral haplotypes persisting across generations (Fig. 2) is concordant with the expected decrease due to genetic drift, and was of similar magnitude in the two populations (*b* = -6 %).

### Real data -Pedigree

Table 1 describes the quality and depth of all pedigrees used in the analyses of real data. At least 6 complete generations were used in each case except for the Spanish breeds, where only one complete generation was available.

**Table 1:** Description of pedigrees

Breed	Population	Ng	Np	MMG (range)	MCG (range)	MEG (range)	Ne
Fleckvieh	-	315	8137	4.7 (0–22)	1.3 (0–6)	2.2 (0–9.1)	239.0
Simmental	-	158	5341	4.3 (0–21)	1.3 (0–7)	2.2 (0–10.5)	174.6
Brown-Swiss	Austria	20	1257	3.9 (0–18)	1.3 (0–6)	2.1 (0–9.7)	124.9
	Germany	54	2677	4.9 (0–20)	1.4 (0–6)	2.4 (0–10.2)	127.6
	Italy	77	3482	4.0 (0–20)	1.3 (0–7)	2.1 (0–9.9)	122.5
	Switzerland	184	5307	4.6 (0–20)	1.5 (0–7)	2.4 (0–10.5)	111.0
	USA	77	1906	4.3 (0–19)	1.5 (0–7)	2.3 (0–10.2)	93.5

Number of genotyped animals (Ng), number of animals in the pedigree (Np), average number of maximum generations (MMG), average number of complete generations (MCG) and average number of equivalent generations (MEG = sum of (1/2)^n^ computed across all known ancestors, where n is the number of generations separating the individual to each known ancestor). Effective sizes (Ne) were computed using the equivalent generations

### Real data—Spanish breeds

In this dataset, a total of 7352 clusters were analysed. Results are presented in Table 2.

**Table 2:** Haplotype results for Spanish breed analyses

Population	Haplotype Variability	Proportion of ancestral haplotypes to total population (%)
Asturiana (as)	41.73	90.88
Avileña (av)	31.84	94.79
Bruna (br)	34.41	93.04
Morucha (mo)	34.82	93.50
Pirenaica (pr)	27.52	94.66
Retinta (re)	30.54	95.86
Rubia Gallega (rg)	31.32	93.33

Average number of haplotypes per cluster across all generations (Haplotype Variability) and the proportion of ancestral haplotypes segregating in the total population across generations

The general haplotype variability (average number of different haplotypes per core across all generations) observed per breed was similar in all cases (average of 33.17) but slightly lower for Pirenaica (27.5) and greater for Asturiana (41.7). Given that only one generation was available (only parent-offspring), a high proportion of ancestral haplotypes were segregating in the total population (93.72 %), and no computation of haplotype similarity across the two generations was performed.

### Real data—Fleckvieh/Simmental populations

A total of 7147 clusters were analysed in the breed populations. Although Fleckvieh presented a greater haplotype variability (71.55) than Simmental (44.98), both breeds showed a similar proportion of segregating ancestral haplotypes in the total population (69.90 % for Fleckvieh and 74.18 % for Simmental).

**Fig. 3:**
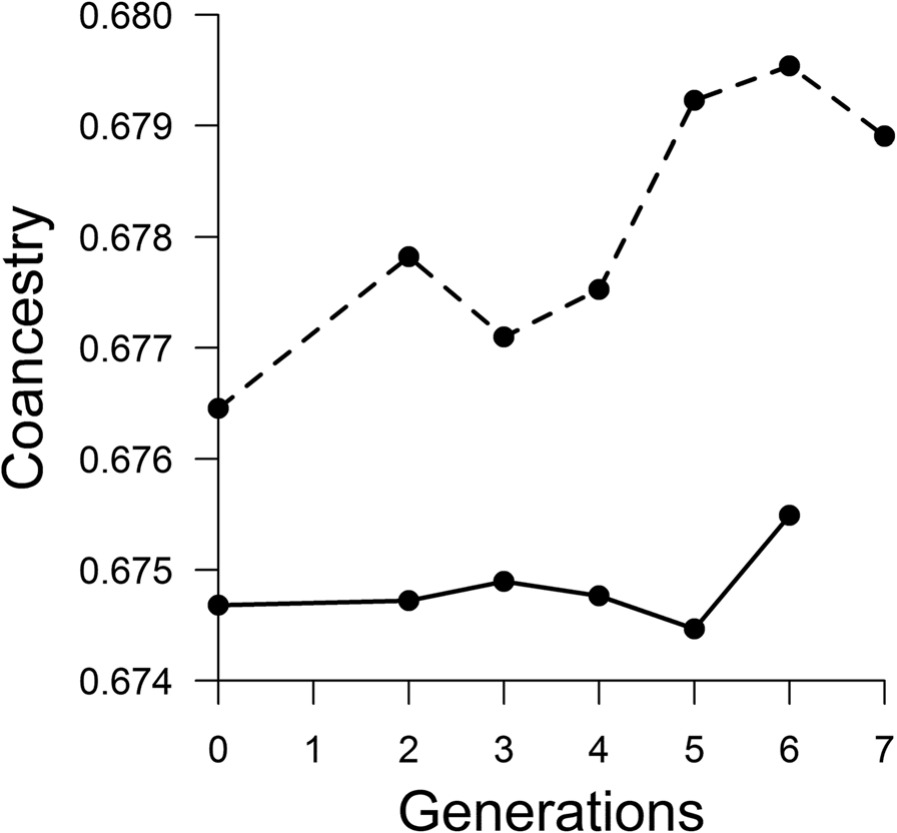
Similarity (co-ancestry) between ancestral haplotypes through generations in Fleckvieh (*solid line*) and Simmental (*dashed line*)

Similarly to the results obtained with simulated data, an increasing trend in the molecular co-ancestry across generations was observed (Fig. 3). The trend was much slower in Fleckvieh (*b*_*A*_ = 7.43×10^-5^) compared to Simmental (*b*_*B*_ = 4.18×10^-4^).

**Fig. 4:**
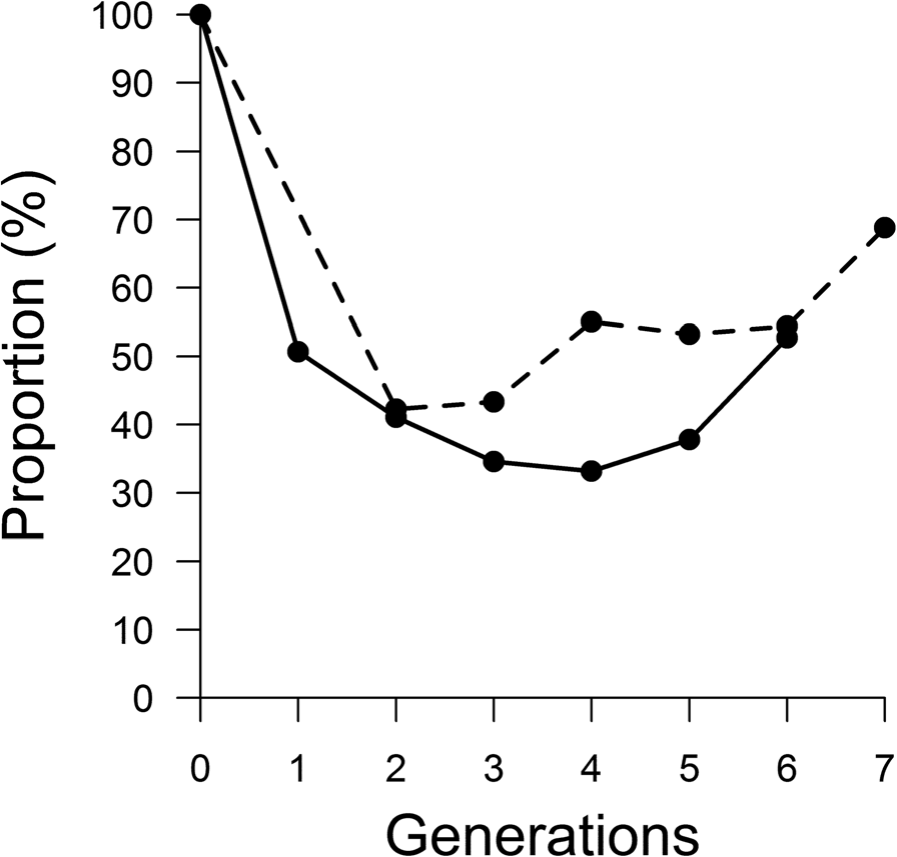
Proportion of ancestral haplotypes segregating per generation in Fleckvieh (*solid line*) and Simmental (*dashed line*)

In Fleckvieh, the change in the proportion of ancestral haplotypes persisting in the subsequent generations (Fig. 4) followed an initial decrease consistent with the results from the simulated data analysis, which may be attributed to strong genetic drift.

An increase in later generations was also observed, which may be a consequence of either the small sample size and/or strong selection leading to an increase in frequency of positively selected haplotypes. Considering only the decrease observed in the first three generations, both breeds showed a similar trend magnitude (*b* = -20 %). However, considering all available generations, the trend diverged between the two breeds, being slower in Simmental (*b*_*B*_ = -2.86 %) than in Fleckvieh (*b*_*A*_ = -6.27 %).

The analysis of ancestral haplotypes across breeds revealed a 13.57 % of the ancestral haplotypes in Simmental also segregating in Fleckvieh across all generations.

### Real data—Brown-Swiss

A total of 7147 clusters were analysed in the five populations. The Swiss population had no genotypes available for the first two pedigree generations and, therefore, the base generation for this population was considered to be generation 2 of the pedigree. Similarly, no genotypes were available for generation 1 of the pedi gree in the Austrian, Italian and German populations.

Table 3 summarises the haplotype variability and the proportion of ancestral haplotypes segregating in each population. Similar values were derived in all populations with the exception of Switzerland, which also presented the highest variability and proportion of ancestral haplotypes, and Austria (lowest variability).

**Table 3:** Haplotype results for Brown Swiss analyses

Population	Haplotype Variability	Proportion of ancestral haplotypes to total population (%)
Austria	11.42	32.46
Germany	22.32	42.45
Italy	21.38	26.65
Switzerland	37.22	66.29
USA	17.90	32.12

Average number of haplotypes per cluster across all generations (Haplotype Variability) and the proportion of ancestral haplotypes segregating in the total population across generations

**Fig. 5:**
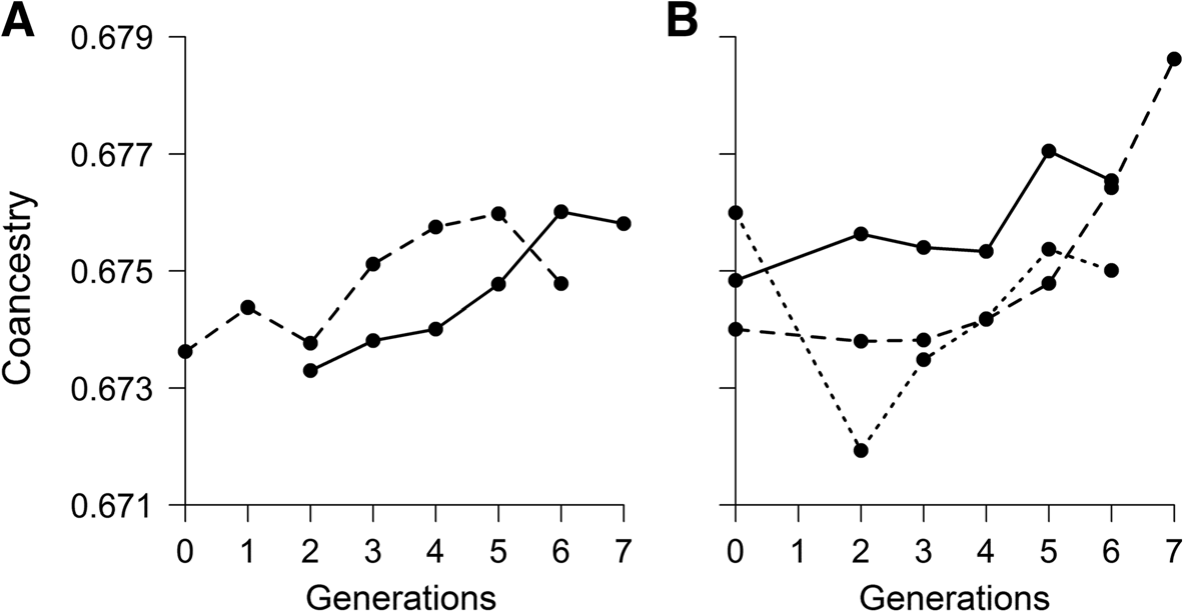
Similarity (co-ancestry) between ancestral haplotypes through generations in Brown-Swiss. **a** forUSA (*dashed line*) and Switzerland (*solid line*) and **b** for non-Swiss European countries: Austria (*solid line*), Italy (*dashed line*) and Germany (*dotted line*)

The progress of the molecular co-ancestry for ancestral haplotypes across generations is shown in Fig. 5, with increasing trends of different magnitude for all countries (*b*_*A*_ = 3.14×10^-4^; *b*_*B*_ = 6.05×10^-4^; *b*_*C*_ = 5.88×10^-5^; *b*_*D*_ = 3.09×10^-4^; *b*_*E*_ = 5.69×10^-4^).

**Fig. 6:**
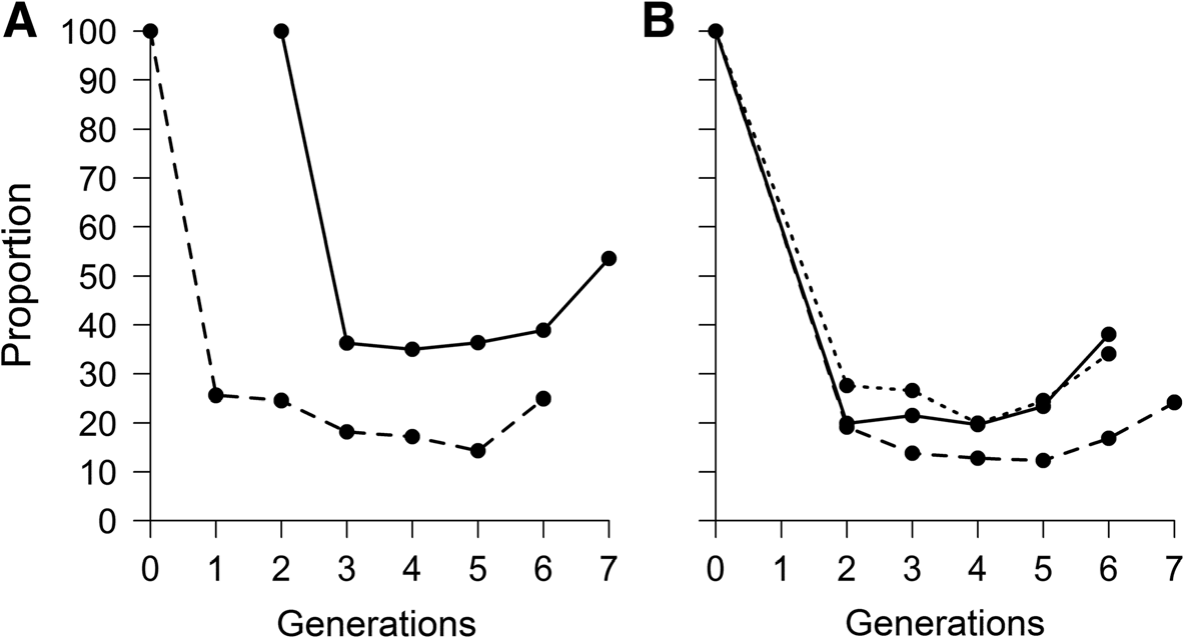
Proportion of ancestral haplotypes segregating per generation in Brown-Swiss. **a** forUSA (*dashed line*) and Switzerland (*solid line*) and **b** for non-Swiss European countries: Austria (*solid line*), Italy (*dashed line*) and Germany (*dotted line*)

As was observed in the simulated and the Fleckvieh/Simmental data analyses, the initial change of the proportion of ancestral haplotypes (Fig. 6) was consistent with the expected decrease due to strong genetic drift emanating from the finite population sizes. Nevertheless, and also in concordance with the results observed in the Fleckvieh/Simmental analysis, small to modest increases were observed in the later generations in all cases. The trend rates in proportion of ancestral haplotypes segregating were similar in all countries except Switzerland, either considering all generations (*b*_*A*_ = -9.15 %; *b*_*B*_ = -8.75 %; *b*_*C*_ = -10.02 %; *b*_*D*_ = -9.11 %) or only the first three generations (*b*_*A*_ = -40.03 %; *b*_*B*_ = -40.41 %; *b*_*C*_ = -36.22 %; *b*_*D*_ = -37.72 %). In the Swiss population, trends were much slower (*b*_*E*_ = -6.36 % and *b*_*E*_ = -32.51 %).

The analyses of shared ancestral haplotypes among populations revealed a close relationship between the USA and Swiss bulls, with a 33.19 % of Swiss ancestral haplotypes segregating in the US population. Lower proportions were shared between the USA population and the other European populations (Italy, Austria and Germany), with an average of 11.58 % ± 1.98 of ancestral USA haplotypes segregating in the latter. Finally, although the Italian, Austrian and German populations shared similar proportions of common ancestral haplotypes (9.33 % ± 1.76 on average), only an average of 5.8 % ± 1.09 of Swiss ancestral haplotypes were segregating in the other populations, thus indicating a lower relationship between them.

## Discussion

The present study has developed and applied a simple procedure to detect ancestral haplotypes based on whole-genome SNP genotypes. The procedure was tested in simulated and real datasets aiming to assess the potential for uncovering the evolutionary history of the populations and detecting common selective patterns among them. Simulated data was designed to mirror the evolutionary process of cattle after the establishment of breed standards, thus providing results that would help understand the outcomes of the real data analyses. Although it would have been of interest to track haplotypes even before the separation of the populations, this would require the availability of older genotypes before the population secession and, in real data, these genotypes are not available.

Higher haplotype variability was observed in the simulated data than in the real datasets because no selection was applied before generation *G0* in the former. On the contrary, genetic drift and selection were present in the real dataset, especially before the oldest available generation (*G0*) of this study, leading to a reduced variability.

Both selection and genetic drift are expected to lead to particular patterns in molecular co-ancestry and frequencies of segregating ancestral haplotypes. In the simulated data, where selection is relatively weak, genetic drift increases the molecular coancestry and reduces the frequencies up to an equilibrium (mutation-drift balance). On the contrary, selection is relatively stronger in the real data, leading to a steadier increase in molecular coancestry due to the combined action of selection and drift. In this case, genetic drift causes an early reduction in the frequencies of ancestral haplotypes but, in later generations, directional selection overcomes the effect of drift. Therefore, the frequencies of positively selected ancestral haplotypes will increase in later generations, leading to slower increases in the average frequency.

Another possible cause of the later increase in frequency observed in the real data could be the effect of a smaller sample size in the most recent generations. However, sample size effects are expected to be random and unbiased and, therefore, frequencies of ancestral haplotypes would be equally likely to increase or decrease. On the contrary, all analysed populations in the real data showed consistent increases, thus supporting the hypothesis of directional selection. Additional evidence of this selection can be obtained from the study of the genomic regions where the most common ancestral haplotypes were segregating in Fleckvieh, Simmental and Swiss-Brown (Additional file 2). These ancestral haplotypes are related (closer than 0.3 Mb) to particular regions in chromosomes 5, 6, 7 and 21 under known selection pressure both in Fleckvieh/Simmental and in Brown-Swiss: Haplotypes located on BTA5 (B*os taurus* Autosome 5) are mainly close to the gene SYT10, which has been related with fitness traits (longevity and maturity) [37] or to the genes PMEL and ERBB3, related to coat colour and facial markings [38]. Similarly, ancestral haplotypes detected on BTA6 correspond to genes related to milk protein and fat percentages such as MEPE, IBSP, LAP3 and MED28 [39] as well as the KIT gene, related to coat colour [38]. In the case of BTA7, no known genes have been yet reported but our ancestral haplotypes are close to previously detected QTLs related to milk production [40] and also close to genes related to pre-ovulatory events (EGR-1), whereas haplotypes in BTA21 contained the gene MEF2A, which has been found to be related to signatures of selection in cattle breeds for milk production [41].

Only two generations (parent-offspring) were available for the Spanish breeds. Therefore, results cannot be conclusive. However, it is noticeable that the most frequent common haplotypes (Additional file 3) were found on autosomes BTA2, BTA7 and BTA11. On BTA2, haplotypes were mainly detected within a region (6–8 Mb) previously found to be associated with a pleiotropic QTL related to marbling, birth weight and calving easy [42] and close to the Myostatin gene (6.2 Mb). On BTA7, haplotypes corresponded to the region found in Fleckvieh that was related to milk production and pre-ovulatory events. On BTA11, haplotypes were detected in a region (66–70 Mb) close to previously detected regions associated with fertility traits in other cattle breeds [43].

Furthermore, the presence of common selective patterns among populations can be confirmed by the existence of frequent ancestral haplotypes segregating in more than one dataset. As example, ancestral haplotypes on BTA6 close to genes associated with protein and fat percentage in milk or genes related to coat colour were detected in both the Fleckvieh/Simmental as well as the Brown-Swiss data. Specifically, regions in BTA6 controlling protein and fat percentage in milk (mainly genes LAP3 and MED28) were detected in all Brown-Swiss populations and in Fleckvieh but not in Simmental. However, no regions common to all breeds and countries were found. This could be the result of a sampling effect and, as only the top five most common haplotypes were studied, there is the possibility that less frequent haplotypes could be segregating in more breeds and countries.

The utility of ancestral haplotype detection is not only limited to the identification of selection signatures. They also provide information about population dynamics and the evolutionary history of different breeds. In the case of the Spanish breeds, the lack of pedigree depth depth in the present study limited the extent of this information. However, recent studies have shown some degree of admixture among these breeds except for Pirenaica, which has been shown to be distanced from other populations [3]. Therefore, in concordance with our results, it was expected that Pirenaica would present a lower genetic variation compared to other breeds, being also concordant with previous studies that show a greater average relatedness in this breed compared to the other Spanish breeds [44].

In the case of the Fleckvieh/Simmental data, at least 6 complete generations were available, providing more information on the segregating patterns of ancestral haplotypes. The Fleckvieh breed was formed in 1830 when Simmental cattle was exported from Switzerland to Germany and Austria in order to improve local breeds. Since then, both Simmental and Fleckvieh have been selected as dual-purpose breeds in similar breeding programmes. Given the close relationship between the breeds, similar signatures of selection could be expected in both. This has led to similar trends in the proportion of ancestral haplotypes segregating through generations. However, given the bottleneck occurring during its origin, a lower haplotype variability and a steadier trend in co-ancestry might have been expected in Fleckvieh. On the contrary, our results showed a higher haplotype variability and a reduced trend in co-ancestry in Fleckvieh when compared to Simmental. Two possible causes could underpin the observed results: i) The enrichment of Simmental haplotypes with haplotypes from local breeds during the establishment and development of the Fleckvieh breed and ii) an unbalanced genetic flow between the two breeds, with strong genetic flow from Simmental into Fleckvieh and a much weaker flow from Fleckvieh to Simmental. Although the first possibility seems more plausible given the relatively small observed proportion of ancestral haplotypes from Simmental segregating in Fleckvieh (only 13.57 % in spite of their expected common selection objectives as dual-purpose breeds), the explanations are not mutually exclusive, and a detailed study would be warranted.

Regarding Brown-Swiss, the breed was originated in Switzerland and exported in 1869 to USA, where it was intensively selected for milk production and then exported back to Europe (Italy, Germany and Austria). Therefore, it is expected that the Swiss population would present a higher variability given the lower selection pressure, while the other populations are expected to have similar levels of variability. However, as no heavy admixture with other breeds is expected, all populations should present similar coancestry (all originated from Switzerland) with maybe Switzerland presenting a slightly lower one (due again to lower selection intensity). The results obtained in the present study reflect these expected dynamics, with the Swiss population showing the highest haplotype variability and the highest proportion of ancestral haplotypes persisting across generations. At the same time, the USA population showed a decrease in both variability and proportion of segregating ancestral haplotypes, which is consistent with the original bottleneck characterised by intensive selection of the imported population. Similar results pertained to the German, Italian and Austrian populations, concordant with their USA origin. In fact, 11.58 % of USA ancestral haplotypes were segregating in these three population compared to the 5.8 % haplotypes of Swiss origin. Although, the Austrian population showed a much lower haplotype variability than other European populations, the proportion of ancestral haplotypes segregating was similar, therefore suggesting that the observed low variability in this population is due to a sample size effect (only 20 Austrian animals were genotyped).

A possible caveat in the present study could be the haplotype size chosen (100 SNPs) when performing the genotype phasing, which should be dependent on the extent of LD across the entire genome. If the haplotype blocks are very small, it is expected an overestimate of the proportion of segregating ancestral haplotypes across time, as small sequences will be easily conserved from one generation to the next. On the contrary, if haplotype blocks are too large, it is expected an underestimate of the proportion of segregating haplotypes, as too large sequences will be rarely conserved from one generation to the next. However, previous studies have already shown that short cores of 100 SNPs similar to the used in this study provide the best phasing results [11]. In future studies, it would be interesting to test different haplotype sizes that could potentially provide information about different selection events. The concept of breed as we know it is very recent, with most breeds being properly established in the last two centuries. Therefore, between species domestication and the formal establishment of breeds there was a period of time where cattle was maintained as a “unique” (non-breed specific) population but with semi-restricted admixture due to geographical and cultural barriers. During that period, animals were likely selected unsystematically according to local preferences and, therefore, ancestral haplotypes linked to these preferences could have been segregating in different breeds at the time of their formation. Old selection signatures related to pre-breed formation would be probably related to small size haplotypes conserved across breeds, whereas most recent selection signatures would be expected to be related to longer haplotypes. In the present study, common selection signatures were found across the breed groups (e.g. in BTA6 related to fat and protein content in milk). With the available information, it is difficult to know if these haplotypes were the result of selection in the pre-breed formation period or directional selection after the breeds were established. Most likely it is a combination of both factors but, given the increase in selection pressure imposed on cattle in the last century, it is more plausible that the increase in frequency of the related haplotypes is mostly the result of recent selection.

Further to the size of the haplotypes, other parameters during phasing and haplotype identification could represent additional considerations. Pedigree information was used in the present study although its effect has been proven to be marginally positive when used in other studies [10]. The length of the core tails is also important, as short tails could lead to a low combinatorial power and, therefore, to false surrogate parents, whereas too long tails would lead to the removal of parents that could have been used as surrogated. Similar issues could rise when stringent error thresholds or a strong overlapping of cores are applied to the identification of surrogates, as too strict parameters would lead to removal of good surrogated parents and, therefore, to a lower number of haplotypes being detected. The parameters used in the present study, with no overlapping but relatively long tails are in accordance with the recommended parameters for the identification of haplotypes proposed by Hickey et al. [11].

Finally, and beyond the scopes of the present study, the detection of ancestral haplotypes can also be used to identify additional important genomic regions for conservation as well as breeding programs. For example, in natural populations under strong natural selection, it is expected that fitness-related genomic regions will be conserved across many generations. Therefore, an approach like the one presented in this study would detect these regions without the need of relevant phenotypes and a follow-up detailed study (e.g. through pathway analysis) could reveal interesting genes located in these regions. Furthermore, studies on livestock populations under artificial selection will reveal genomic regions associated with the breeding goal traits that have been maintained across generations by selection (as shown in this study), thereby leading to the identification of genes of interest (e.g. in Gene Assisted Selection) [45]. Additional examples can be related to other relevant genomic regions: for example, it is expected that genomic regions with a high haplotype variability across different populations and breeds could be indicative of possible recombination hotspots.

## Conclusions

This study presents a simple procedure to detect ancestral haplotypes in cattle populations. Signatures of selection were detected in various cattle breeds, demonstrating potential to uncover the population dynamics of these breeds. The existence of common selective goals across breeds is concordant with the detection of common segregating haplotypes and the increase in frequency of some ancestral haplotypes being selected through generations. Furthermore, the evolutionary history of the studied populations is mirrored by the population specific patterns of variability, frequency and co-ancestry of ancestral haplotypes, thus reflecting not only the evolution of each particular population but also the relationship among them.

## Abbreviations

BTA, *Bos taurus* autosome; IBD, identity by descent; LD, linkage disequilibrium; QTL, quantitative trait loci; SNP, single nucleotide polymorphisms

## Additional files

Additional file 1: QC summary performed for real datasets. As the Spanish breeds dataset was provided by other study, no information can be found on the original number of animals. (XLSX 12 kb) Additional file 2: Five most common ancestral haplotypes per breed/population in Fleckvieh, Simmental and Brown-Swiss. Information presented for each haplotype (Hap) pertain to chromosome (Chr), genomic coordinates of the haplotype (starting SNP and ending SNP base pair positions), frequency (as provided by AlphaPhase), relative frequency to other haplotypes (frequency of the haplotype divided by the sum of the frequencies of all haplotypes detected in the cluster for the total population), genes of interest (closer than 0.3 Mb of the haplotype) and closest gene with its coordinates. Coordinates are given according to the Genome assembly UMD3.1. (XLSX 14 kb) Additional file 3: Five most common ancestral haplotypes per breed in the Spanish breeds dataset. Information presented for each haplotype (Hap) pertain to chromosome (Chr), genomic coordinates of the haplotype (starting SNP and ending SNP base pair positions), frequency (as provided by AlphaPhase) and relative frequency to other haplotypes (frequency of the haplotype divided by the sum of the frequencies of all haplotypes detected in the cluster for the total population). Coordinates are given according to the Genome assembly UMD3.1. (XLSX 13 kb)
